# Supplementary material for: Empirical methods for controlling false positives and estimating confidence in ChIP-Seq peaks
Source: BMC Bioinformatics. 2008 Dec 5;9:523. doi: 10.1186/1471-2105-9-523 (PMC2628906; doi:10.1186/1471-2105-9-523)
Supplement: Additional File 1 — A variety of html documents from the USeq web site detailing the available applications, their best usage, output file type descriptions, command line menus, etc. [file 1471-2105-9-523-S1.zip › USeqUserGuides/JavaDoc.zip!/JavaDoc/trans/misc/class-use/GrGraph.html]

Uses of Class trans.misc.GrGraph


|  |  |  |
| --- | --- | --- |
| **Overview** \| **Package** \| **Class** \| **Use** \| **Tree** \| **Deprecated** \| **Index** \| **Help** | |  |
| PREV   NEXT | **FRAMES**    **NO FRAMES**     **All Classes** |  |


---


## **Uses of Class trans.misc.GrGraph**

| Packages that use GrGraph | |
| --- | --- |
| **trans.misc** | Rough working scripts. |
| **trans.roc** | For manipulating the BAC dmel spike in data and generating ROC curves. |

Uses of GrGraph in trans.misc

| Methods in trans.misc that return GrGraph | |
| --- | --- |
| `static GrGraph[]` | `GrGraph.loadSgrFile(java.io.File sgrFile)`             Loads an sgr file returning chromosome specific GrGraphs. |
| `static GrGraph[]` | `Wig2Bar.parseFixedStepWigFile(java.io.File wigFile, float skipValue)`             Parses a fixedStep wig file, skips any track type data. |
| `static GrGraph[]` | `Wig2Bar.parseVariableStepWigFile(java.io.File wigFile, float skipValue)`             Parses a variableStep wig file, skips any track type data. |
| `static GrGraph` | `Util.readSimpleGrBarFile(java.io.File simpleBarFile)`             This is a complete hack of Gregg Helt's IGB code to parse bar files. |

| Methods in trans.misc with parameters of type GrGraph | |
| --- | --- |
| `static boolean` | `Util.writeSimpleBarFile(GrGraph gr, java.io.File barFile)`             Writes a simple bar format graph for a xxx.gr type graph. |

Uses of GrGraph in trans.roc

| Methods in trans.roc that return GrGraph | |
| --- | --- |
| `static GrGraph[]` | `Sgr.splitByChrom(Sgr[] sortedSgrs)`             Splits a Sgr[] by chromosome. |

| Methods in trans.roc with parameters of type GrGraph | |
| --- | --- |
| `static Gr[]` | `Gr.parseGrGraph(GrGraph gr)` |

---


|  |  |  |
| --- | --- | --- |
| **Overview** \| **Package** \| **Class** \| **Use** \| **Tree** \| **Deprecated** \| **Index** \| **Help** | |  |
| PREV   NEXT | **FRAMES**    **NO FRAMES**     **All Classes** |  |


---
